# Synthesis, Characterisation and Preliminary Antimicrobial Evaluation of Chitosan-4-Anisaldehyde Conjugates

**DOI:** 10.3390/polym17223017

**Published:** 2025-11-13

**Authors:** Danelya N. Makhayeva, Dayana D. Mukhamediya, Saiyara R. Tairova, Ardak Jumagaziyeva, Galiya S. Irmukhametova, Vitaliy V. Khutoryanskiy

**Affiliations:** 1 Faculty of Chemistry and Chemical Technology, Al-Farabi Kazakh National University, Almaty 050040, Kazakhstan; makhayeva.danelya@kaznu.kz (D.N.M.); mukhamediya@daniellipharm.kz (D.D.M.); sayataiko1592@icloud.com (S.R.T.);; 2 Scientific Centre for Anti-Infectious Drugs, Almaty 050060, Kazakhstan; r_dawa@mail.ru; 3 Reading School of Pharmacy, University of Reading, Whiteknights, Reading P.O. Box 224, UK

**Keywords:** chitosan, Schiff base formation, 4-anisaldehyde, antimicrobial properties, gels

## Abstract

The growing need for effective antimicrobial polymeric materials has prompted extensive development of functional chitosan derivatives with enhanced physicochemical and biological properties. In this work, the conjugates of chitosan with 4-anisaldehyde (ChT-AA) were synthesised through Schiff base formation at various molar ratios and characterised using FT-IR, ^1^H NMR, and thermal analysis techniques (TGA/DSC). The spectral data confirmed the successful formation of imine (C=N) linkages and the incorporation of aromatic anisaldehyde fragments into the chitosan structure. Thermal analysis demonstrated increased stability and a higher glass transition temperature for ChT-AA compared with native chitosan, indicating reduced polymer chain mobility and enhanced structural rigidity. Viscoelastic gels based on the synthesised ChT-AA (1:3) and methylcellulose were prepared and evaluated for their rheological properties and antimicrobial performance. Rheological studies revealed non-Newtonian shear-thinning behaviour of these gels with pronounced thixotropy, confirming reversible network recovery after shear deformation. Antimicrobial evaluation of chitosan, its 4-anisaldehyde conjugate (ChT–AA, 1:3), and free 4-anisaldehyde revealed distinct activity patterns. The gels showed no inhibition in the disk diffusion assay, likely due to limited diffusion of the active components. In liquid media, both ChT and ChT–AA exhibited identical minimum inhibitory concentrations (MICs) against *E. coli* (0.313 mg/mL) and *C. albicans* (1.250 mg/mL), whereas ChT–AA showed two-fold stronger activity against *S. aureus* (0.313 mg/mL vs. 0.625 mg/mL for ChT). Free 4-anisaldehyde was most active against *S. aureus* (MIC = 0.175 mg/mL) but less effective against the other strains, confirming its narrower spectrum. These results indicate moderate antimicrobial efficacy in solution but limited activity in gel form due to restricted diffusion.

## 1. Introduction

The growing threat of antimicrobial resistance and the increasing incidence of device- and wound-associated infections highlight an urgent need for novel antimicrobial polymeric materials that combine efficacy, safety, and biocompatibility [1,2]. Conventional antibiotics are becoming less effective, and polymer-based materials with intrinsic or functionalized antimicrobial properties are gaining attention as sustainable alternatives to combat resistant microorganisms. Among these, gel formulations have emerged as particularly promising systems due to their high water content, permeability, and ability to provide a moist environment favourable for tissue healing [3,4,5].

Chitosan (ChT) is a cationic polysaccharide obtained by deacetylation of chitin, one of the most abundant biopolymers found in the exoskeletons of crustaceans and fungal cell walls. Due to its biocompatibility, biodegradability, non-toxicity, and film-forming properties, chitosan has gained significant attention in biomedical, pharmaceutical, and food applications [6,7]. However, its poor solubility in neutral and basic media, as well as its limited antimicrobial activity in certain environments, restrict its broader applications [8].

Chemical modification of chitosan is an effective strategy to enhance its physicochemical and biological properties. Among various modification routes, the formation of Schiff bases via the condensation of chitosan’s amino groups with aldehydes or ketones is particularly attractive, as it improves its solubility, metal-chelating ability, and biological activity [9,10,11]. For instance, Schiff bases of chitosan synthesised using various aromatic aldehydes have previously demonstrated improved antibacterial and antifungal activity while retaining low cytotoxicity [12]. Furthermore, vanillin–chitosan Schiff-base-stabilised emulsions exhibited confirmed structural conjugation, pH-responsive behaviour, and antibacterial effects against *S. aureus* and *E. coli*, supporting the potential of such systems as smart antimicrobial materials [13]. Schiff bases derived from natural aldehydes (cinnamaldehyde, benzaldehyde, vanillin, citronellal, and citral) are especially promising due to their inherent bioactivity and reduced toxicity compared to synthetic analogues [14].

4-Anisaldehyde (AA) is a naturally occurring aromatic aldehyde found in essential oils of anise (*Pimpinella anisum*) and fennel (*Foeniculum vulgare*). It has antimicrobial, antioxidant, and anti-inflammatory properties [15,16]. Its conjugation with chitosan through Schiff base formation may confer synergistic bioactivity, yielding materials with enhanced antimicrobial performance and potential biomedical applications [17].

Gels based on chitosan and its derivatives are widely explored as wound dressings, drug delivery matrices, and scaffolds for tissue engineering due to their high water-retention capacity, porosity, and biocompatibility. Incorporating bioactive Schiff base moieties into chitosan gels can further enhance their antimicrobial and physicochemical behaviour, which is crucial for preventing infections and promoting tissue regeneration [18].

Gels formulated from chitosan derivatives have attracted considerable attention in this context due to their excellent water-retention capacity, porosity, and biocompatibility, making them potentially suitable for use as wound dressings, drug delivery systems, and tissue engineering scaffolds [19]. In the present study, gels were developed using 4-anisaldehyde-modified chitosan as a functional component and methylcellulose as a gelling agent. The incorporation of bioactive Schiff base functionalities into the chitosan structure, combined with the structural stability and swelling behaviour imparted by methylcellulose, is expected to enhance the antimicrobial performance of the resulting materials.

In this study, chitosan-4-anisaldehyde Schiff bases were synthesised and characterised using Fourier-transform infrared spectroscopy (FTIR), ^1^H NMR spectroscopy, rheological measurements and thermal analysis. Furthermore, gels based on these derivatives were prepared, and their antimicrobial activities were systematically evaluated against selected bacterial and fungal strains.

## 2. Materials and Methods

### 2.1. Materials

4-anisaldehyde (AA, 98%) (Sigma Aldrich, Bangalore, India), chitosan of low molecular weight (ChT) (degree of deacetylation is 75–85%) (Sigma Aldrich, Shanghai, China), methylcellulose-40 kDa (viscosity 400 cP) (Sigma Aldrich, St. Louis, MO, USA), lactic acid (80%) (Kupavnareaktiv, Staraya Kupavna, Moscow region, Russia), and fluorescein sodium salt (NaFl) (Sigma Aldrich, Hamburg, Germany), sodium hydroxide (NaOH, Labchimprom, Almaty, Kazakhstan), and ethyl alcohol (90%, “Talgar-Spirit” JSC, Almaty region, Kazakhstan) were used in this study without further purification.

### 2.2. Synthesis of Chitosan-4-Anisaldehyde Conjugate (ChT-AA)

The synthesis of ChT-AA was performed according to the method reported in [20] with some changes. Chitosan (1 g) was dissolved in 50 mL of 2% lactic acid solution and stirred at room temperature for 6 h. Subsequently, ethanol solutions of 4-anisaldehyde were prepared at different molar ratios relative to chitosan (1.6:1, 1:3, and 1:1), as summarised in Table 1. The corresponding volume of AA solution was added dropwise (10 mL) to the chitosan solutions. The mixtures were stirred for an additional 6 h at 50 °C.

These suspensions were slowly poured into a 5% (*w*/*v*) NaOH solution, resulting in the immediate precipitation of the conjugates. The precipitates were collected by vacuum filtration using a Büchner funnel, then washed successively thoroughly six times with deionised water and ethanol, to remove unreacted aldehyde and residual acid. The purified solids were frozen and lyophilised for 24 h to yield white powders of the ChT-AA conjugates.

### 2.3. Preparation of Gels Containing ChT-AA Conjugates and Chitosan

The conjugate [ChT-AA] (1:3) and chitosan (0.2 g) were each dissolved in 10 mL of 2% (*v*/*v*) lactic acid under magnetic stirring until homogeneous solutions were obtained. Separately, an aqueous solution of methylcellulose (7.5% *w*/*v*) was prepared by gradually adding the polymer to hot water under continuous stirring until complete dissolution. The chitosan (or ChT-AA) and methylcellulose solutions were then combined in a 1:2 volume ratio and mixed thoroughly to obtain a uniform gel matrix. The final concentration of conjugate [ChT-AA] (1:3) and chitosan in gel is 0.67% *w*/*v*, and for methylcellulose is 5% *w*/*v*. The pH of the resulting gel was adjusted to 5.0 by the dropwise addition of 0.5 M NaOH solution.

### 2.4. Physicochemical Characterisation

*FTIR spectroscopy.* Fourier-transform infrared (FTIR) spectra of the synthesised and dried conjugates were recorded in the range of 500–4500 cm^−1^ using a Vertex 70 V spectrometer (Bruker, Ettlingen, Germany) equipped with a germanium ATR crystal and OPUS 7.2.139.1294 software.

*^1^H NMR spectra* of chitosan and its derivatives (ChT-AA) were recorded using a JNM-ECA spectrometer (500 MHz, Boston, MA, USA). Samples were dissolved in deuterated water (D_2_O) with the addition of 2–3 drops of trifluoroacetic acid.

The degree of substitution (DS) was determined for the synthesised conjugates by dividing the integration intensity of the aromatic proton peaks (δH 6.7–7.9 ppm) by the integration intensity of the chitosan glucosamine ring protons (H2–H6, δH 3.2–4.2 ppm), as shown in Equation (1). This ratio reflects the relative number of substituted 4-anisaldehyde units per glucosamine residue, allowing accurate estimation of the substitution degree based on ^1^H NMR data.

(1) $$\mathrm{DS}=(\frac{I(Ar-H)/4}{I(H2-H6)/6})\times 100\%$$

*Thermogravimetric analysis (TGA).* TGA measurements were performed on an SKZ1053A thermogravimetric analyser (SKZ International Co., Ltd., Jinan, Shandong, China) using approximately 8 mg of dry sample. The heating was carried out at a rate of 10 °C/min in the temperature range of 25–600 °C under a nitrogen atmosphere, using ceramic crucibles.

*Differential scanning calorimetry (DSC).* DSC measurements were performed on an SKZ1052 DSC instrument (SKZ International Co., Ltd., Jinan, Shandong, China) using approximately 8 mg of sample. The heating was conducted at 10 °C/min in the range of 30–150 °C under an air atmosphere, employing Tzero™ aluminium pans. Data acquisition and analysis were carried out using DSC Thermal Analysis software (version 2023051113_s).

### 2.5. Rheological Measurements

The rheological properties of the gels were investigated using a Lamy Rheology RM 200 viscometer (Lamy Rheology Instruments, Champagne-au-Mont-d’Or, France). Measurements were performed at 25 °C with spindle speeds ranging from 0.5 to 800 rpm, employing an R3-disk spindle (L-3 system, diameter 34.69 mm). The gel sample volume was 30 mL. The obtained data were used to determine shear stress, shear rate, and viscosity, and to plot flow and viscosity curves for comprehensive characterisation of the rheological behaviour of the gels.

The analysis was conducted using 17 sequentially increasing spindle speeds (rpm) in ascending mode. To investigate structural breakdown, the spindle was rotated at a maximum speed of 800 rpm for 10 s, followed by a resting period of 10 min [21]. Subsequently, viscosity values (mPa·s) were recorded for each of the 17 spindle speeds during the decreasing stage (descending mode). All rheological measurements were performed in triplicate, and the data are presented as mean ± SD. The resulting ascending and descending flow curves were compared to evaluate the thixotropic behaviour of the gels. To improve readability, SD bars were omitted on the hysteresis plot; however, the full data, including mean ± SD, are provided in the Results section.

The evaluation of thixotropic behaviour was carried out using the experimental data obtained during ascending and descending shear modes. Shear rate (γ, s^−1^) was calculated according to Equation (2).

(2) $$\dot{\gamma }=K\cdot \mathrm{RPM},$$

where K is the spindle constant and RPM is the spindle speed. For our R3-disk spindle K = 1 [22].

Shear stress (τ, mPa) was determined as

τ = η·γ,(3)

where η is viscosity (mPa·s). The difference between the ascending and descending flow curves was used to characterise the thixotropic behaviour of the gels.

### 2.6. Antimicrobial Activity

The antimicrobial activity against *Staphylococcus aureus*, *Escherichia coli* and *Candida albicans* was evaluated by the disk diffusion method at the Scientific Centre for Anti-Infective Drugs (Almaty city, Kazakhstan), in the Laboratory of Microbiology. General antimicrobial assessment was carried out using the disk diffusion method on Mueller-Hinton agar (for bacteria) and Sabouraud agar (for yeast fungi). Reference strains *Escherichia coli* (ATCC 8739), *Staphylococcus aureus* (ATCC 6538), and *Candida albicans* (ATCC 90028) were used as test organisms.

Agar plates were inoculated with microbial suspensions adjusted to 0.5 McFarland units (~1.5 × 10^8^ CFU/mL). Sterile paper disks (6 mm in diameter), pre-impregnated with the gel compositions (1 mg/mL), were placed on the surface. For this method, four gel formulations were prepared: methylcellulose gel (MC), chitosan–methylcellulose gel (MC–ChT), conjugate gel of chitosan–anisaldehyde with methylcellulose (MC–ChT–AA), and methylcellulose gel containing free anisaldehyde (MC–AA). Plates were incubated at 37 °C for 24 h for bacteria and 48 h for fungi. Growth inhibition zones were measured in millimetres. All experiments were performed in triplicate, and mean values with standard deviations were calculated to ensure statistical reliability and reproducibility.

The antimicrobial activity was evaluated using a two-fold serial broth microdilution method according to CLSI guidelines [23,24,25]. Tests were performed in sterile 96-well plates with Müller-Hinton broth or Sabouraud for *Candida albicans*. Serial dilutions of ChT (10 mg/mL), ChT + AA (10 mg/mL), 4-anisaldehyde (2.5 μL/mL), and solvent control (1% lactic acid + 1% DMSO) were prepared in triplicate. Bacterial suspensions adjusted to 0.5 McFarland (~1.5 × 10^8^ CFU/mL) were diluted to ~1.5 × 10^6^ CFU/mL, and 10 μL of inoculum was added to each well. Plates were incubated at 37 °C for 24 h, then treated with 0.05 mL of 0.05% resazurin and incubated for 30 min. The minimum inhibitory concentration (MIC) was recorded as the lowest sample concentration preventing colour change (blue → pink), indicating inhibition of microbial growth.

## 3. Results

Chitosan derivatives have found applications in various fields of biomedicine and pharmaceuticals due to their unique structural features and biological properties [26]. An important property of chitosan is its antibacterial activity. In this study, chitosan was conjugated with 4-anisaldehyde, another antimicrobial compound, to explore potential synergistic effects.

**Figure 1:**
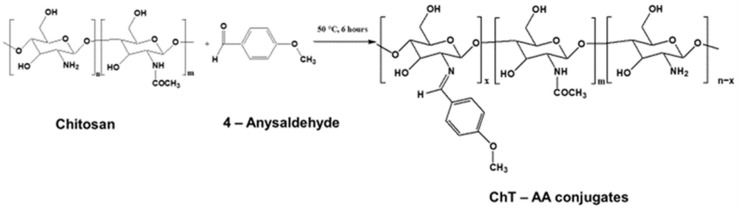
Reaction scheme for the synthesis of the Schiff base derived from chitosan and 4-anisaldehyde (ChT-AA).

The ChT-AA conjugates were synthesised at varying molar ratios of chitosan to AA (1.6:1, 1:1, 1:2, and 1:3), using 2% lactic acid solution and ethanol as solvents. Figure 1 shows the reaction scheme resulting in imine (C=N) bond formation between the amino groups of chitosan and the carbonyl groups of the aldehyde. The formation of the conjugates was confirmed using FTIR and ^1^H NMR spectroscopy.

**Figure 2:**
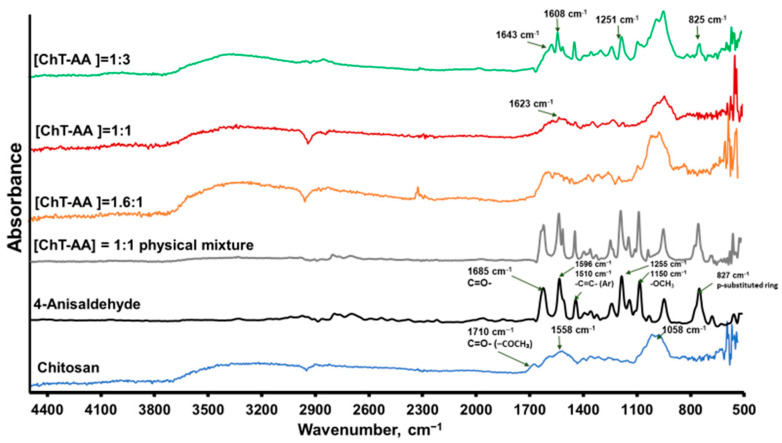
FTIR spectra of chitosan, 4-anisaldehyde, and ChT–AA conjugates at various molar ratios.

The FT-IR spectra presented in Figure 2 demonstrate the structural changes in chitosan upon conjugation with 4-anisaldehyde at different molar ratios. Pure chitosan shows its characteristic absorption bands at ~3270 cm^−1^ (O–H and N–H stretching), 2920–2870 cm^−1^ (C–H stretching), and a broad region between 1550–1650 cm^−1^ assigned to amide I (C=O) and N–H bending vibrations. In addition, the weak band at 1710 cm^−1^ corresponds to the C=O stretching of residual acetyl groups from incomplete deacetylation.

Upon increasing anisaldehyde content, new absorption features appear at 1623–1643 cm^−1^, attributable to imine (C=N) stretching, accompanied by a noticeable decrease in N–H deformation intensity—indicating partial consumption of amino groups and Schiff-base formation between the aldehyde carbonyl and chitosan amines. To ensure that these changes are not caused merely by hydrogen bonding or physical adsorption, control spectra of 4-anisaldehyde, pure chitosan, and their physical mixture were compared (Figure 2). The ChT–AA(1:3) conjugate displays additional peaks at 1608–1591 cm^−1^ (aromatic C=C), 1251–1255 cm^−1^ (methoxy C–O–C), and 825–827 cm^−1^ (C–H of a para-substituted aromatic ring), which are absent or much weaker in the physical mixture. These distinct bands verify the successful chemical incorporation of anisaldehyde moieties into the chitosan backbone, confirming that covalent imine bond formation, rather than simple physical interaction, predominates in the ChT–AA conjugates.

**Figure 3:**
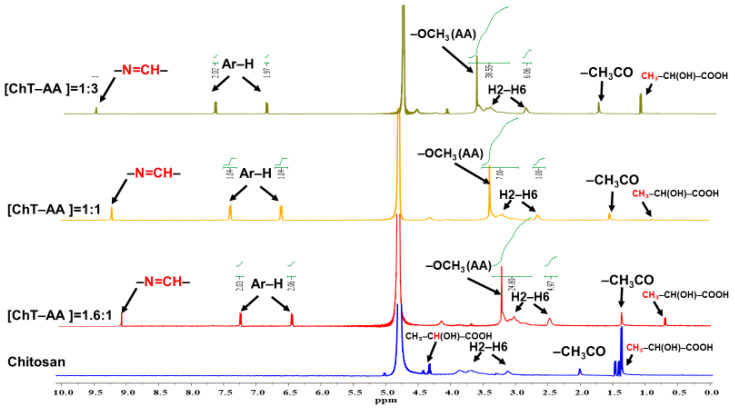
^1^H NMR spectra of pure chitosan and its Schiff base derivatives with 4-anisaldehyde (ChT-AA) at different molar ratios. The green lines represent the integration curves of the characteristic proton signals.

The ^1^H NMR spectra (Figure 3) of pure chitosan and its conjugates with 4-anisaldehyde confirm the successful formation of Schiff base structures.

In the chitosan spectrum, the characteristic resonances of the glucosamine backbone are observed at δH 3.2–4.2 ppm (H2–H6) and δH 4.7–5.0 ppm (H1), while a small peak at δH 1.9–2.1 ppm corresponds to residual N-acetyl groups. This spectrum is broadly consistent with previously reported ^1^H NMR data for pure chitosan [27], except for several additional signals observed at 3.6–4.1 ppm and 1.25–1.50 ppm, which are attributed to lactic acid residues (CH_3_–CH(OH)–COOH), which originate from lactic acid used as the solvent for chitosan dissolution.

In contrast, the spectra of ChT–AA samples exhibit new signals at δH 9.1–9.3 ppm, assigned to the imine proton (–CH=N–), confirming Schiff base formation between chitosan and 4-anisaldehyde. The aromatic proton signals (Ar-H) appear at δH 6.9–7.9 ppm, while a singlet at δH 3.8–3.9 ppm corresponds to the –OCH_3_ group of the 4-anisaldehyde fragment.

The gradual increase in the intensity of imine, aromatic, and –OCH_3_ signals with higher 4-anisaldehyde content indicates an increased degree of substitution. Thus, the appearance of –CH=N–, Ar–H, –OCH_3_, and lactic acid resonances provides strong evidence of successful conjugation, consistent with the FTIR results.

The relative degrees of substitution (DS) calculated from the ^1^H NMR spectra were approximately 21% for the [ChT–AA] = 1.6:1, 40% for the [ChT–AA] = 1:3, and 13.1% for the [ChT–AA] = 1:1 samples.

**Figure 4:**
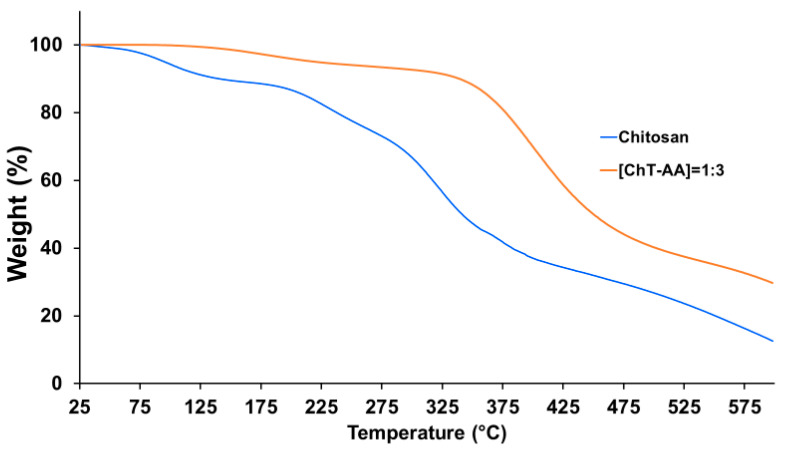
TGA curves of pure chitosan and its conjugate with 4-anisaldehyde (ChT-AA).

Further characterisation of chitosan and its conjugate with 4-anisaldehyde ([ChT-AA] = 1:3) was conducted using thermal gravimetric analysis (Figure 4). The first stage, observed below approximately 120 °C, involves a minor weight loss of about 10%, corresponding to the evaporation of physically adsorbed and bound water. In contrast, the [ChT-AA] = 1:3 sample exhibits a substantially lower weight loss (~0.5%) in this region, likely due to the decreased hydrophilicity of the modified polymer arising from the incorporation of hydrophobic aromatic fragments. The main decomposition of the polysaccharide backbone occurs in the range of 200–350 °C for chitosan, with a residual weight of ~66% at 300 °C (weight loss ~34%). This is in agreement with the previous studies of chitosan [24]. For [ChT-AA] = 1:3, the initial weight loss in the range of 200–320 °C is lower (~20–25%), and the main chain decomposition shifts to a higher temperature interval (320–450 °C), indicating improved thermal stability of the modified sample. At 500 °C, the residual weight of chitosan is ~26.7%, whereas [ChT-AA] = 1:3 retains ~40.3%.

These results clearly demonstrate that chemical modification of chitosan enhances its thermal stability, which can be attributed to the formation of imine (C=N) linkages and the introduction of rigid aromatic fragments that strengthen the polymer structure.

**Figure 5:**
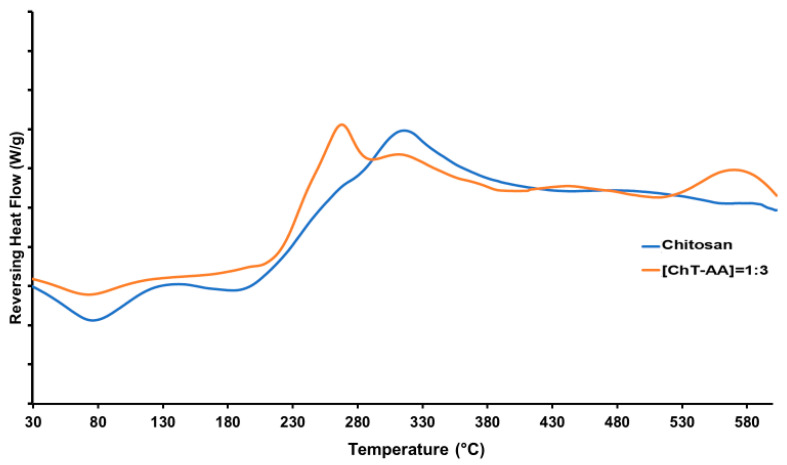
DSC curves of pure chitosan and its Schiff base derivative with anisaldehyde (ChT-AA).

DSC was employed to investigate the thermal transitions of pure chitosan and its conjugate with anisaldehyde ([ChT-AA] = 1:3) (Figure 5). For pure chitosan, a glass transition temperature (T_g_) was detected at approximately 117 °C, which is in agreement with literature data for this polysaccharide [28]. In contrast, the ChT-AA conjugate exhibited a slightly higher T_g_ of 122.5 °C, indicating restricted segmental mobility of the macromolecular chains as a result of Schiff base formation. The increase in T_g_ can be attributed to the presence of rigid imine bonds (C=N) and bulky anisaldehyde aromatic fragments introduced into the polymer backbone, which enhance intermolecular interactions and reduce chain flexibility.

In addition to T_g_, both samples exhibited an endothermic event within the range of 70–100 °C (at 76 °C for chitosan and 74 °C for ChT-AA), attributed to the release of residual bound water. At higher temperatures, an exothermic decomposition peak appeared at approximately 314 °C for chitosan and 309 °C for ChT-AA, corresponding to the degradation of glucosamine units [29]. Notably, the ChT-AA sample exhibited a slight shift of this decomposition event toward a slightly lower temperature and reduced intensity, indicating a more compact structure with restricted chain mobility and overall improved thermal stability. Table 2 summarises the results of the thermal analysis of all samples.

The FTIR, ^1^H NMR, and thermal analyses consistently confirm the successful Schiff base formation between chitosan and 4-anisaldehyde. The results demonstrate improved thermal stability and increased glass transition temperature (T_g_), reflecting enhanced structural rigidity.

### 3.1. Rheological Analysis

**Figure 6:**
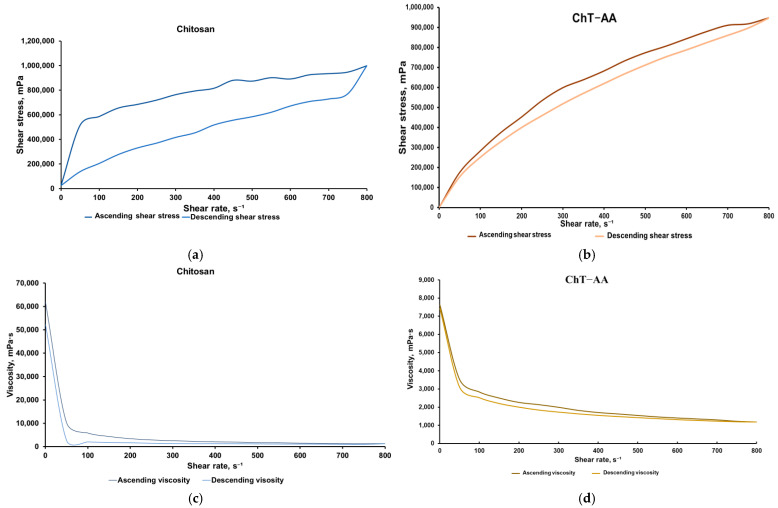
Results of the rheological analysis of gels containing chitosan and ChT-AA: Flow curves of chitosan (**a**) and ChT-AA containing gels (**b**); Viscosity curves of chitosan (**c**) and ChT-AA containing gels (**d**).

Chitosan and its conjugates were formulated into gels by mixing them with methylcellulose, a well-known gelling agent. Since these gels were intended for antimicrobial applications, characterisation of their rheological properties was of particular interest, as it influences their ability to spread over surfaces and their subsequent retention. The rheological behaviour of gels containing chitosan and its conjugate with 4-anisaldehyde was evaluated using flow (shear stress vs. shear rate, Figure 6a) and viscosity (viscosity vs. shear rate, Figure 6b) curves.

Both systems exhibited typical non-Newtonian shear-thinning behaviour, where shear stress increased nonlinearly with shear rate, and viscosity decreased sharply at low shear rates before reaching a plateau at higher rates. The gel containing unmodified chitosan exhibited higher viscosity and shear stress values across the entire shear rate range, indicating a denser and more stable gel structure. In contrast, the gel with ChT-AA showed significantly lower viscosity and shear stress, indicating an increased flowability of this formulation. These differences in rheological behaviour demonstrate variations in the internal organisation of the gels and confirm the more pronounced pseudoplastic characteristics of the ChT-AA system.

The presence of a hysteresis loop between the ascending and descending branches confirms the thixotropic character of both gels, where network breakdown occurs under applied shear and partial recovery follows upon shear reduction. For the gel containing [ChT-AA] 1:3, viscosity decreased from ~7630 mPa·s at low shear (0.5 rpm) to ~1185 mPa·s at 800 rpm, while shear stress steadily increased to ~948,000 mPa. The minimal divergence between ascending and descending curves indicates a narrow hysteresis loop, reflecting efficient structural regeneration and stable reversible thixotropic behaviour. Table 3 presents the mean values of shear stress and viscosity for ascending and descending curves of both gels.

### 3.2. Antimicrobial Properties of Chitosan, ChT-AA, and Corresponding Gels

Chitosan, as a polycationic biopolymer, exhibits antimicrobial activity largely due to its cationic nature [30]. This property forms the basis for its wide application as an antimicrobial agent. Extensive studies, including both in vivo and in vitro experiments, have investigated its antimicrobial potential against a variety of microorganisms, including fungi and bacteria [31]. Chitosan has demonstrated effectiveness against both Gram-positive and Gram-negative bacteria. Examples of Gram-positive bacteria sensitive to chitosan include *Staphylococcus epidermidis*, *Streptococcus mutans*, *S. aureus*, and *E. faecium*, while Gram-negative bacteria such as *E. coli*, *P. aeruginosa*, and *Salmonella typhimurium* have also shown susceptibility. The antifungal activity of chitosan and its derivatives has been demonstrated against several fungal strains, including *C. albicans*, *Fusarium solani*, *Candida glabrata*, *Trichophyton equinum*, and *Candida krusei* [32].

4-Anisaldehyde (AA) is a trans-isomer of anisole, primarily extracted from *Pimpinella anisum* L. The U.S. Food and Drug Administration (FDA) has classified 4-anisaldehyde as “Generally Recognised as Safe” (GRAS) for use as a natural additive in food products [33]. Previous studies have shown that AA can inhibit the growth of *Candida albicans*, *Staphylococcus aureus*, *Listeria monocytogenes*, and *Pseudomonas aeruginosa* [34].

**Figure 7:**
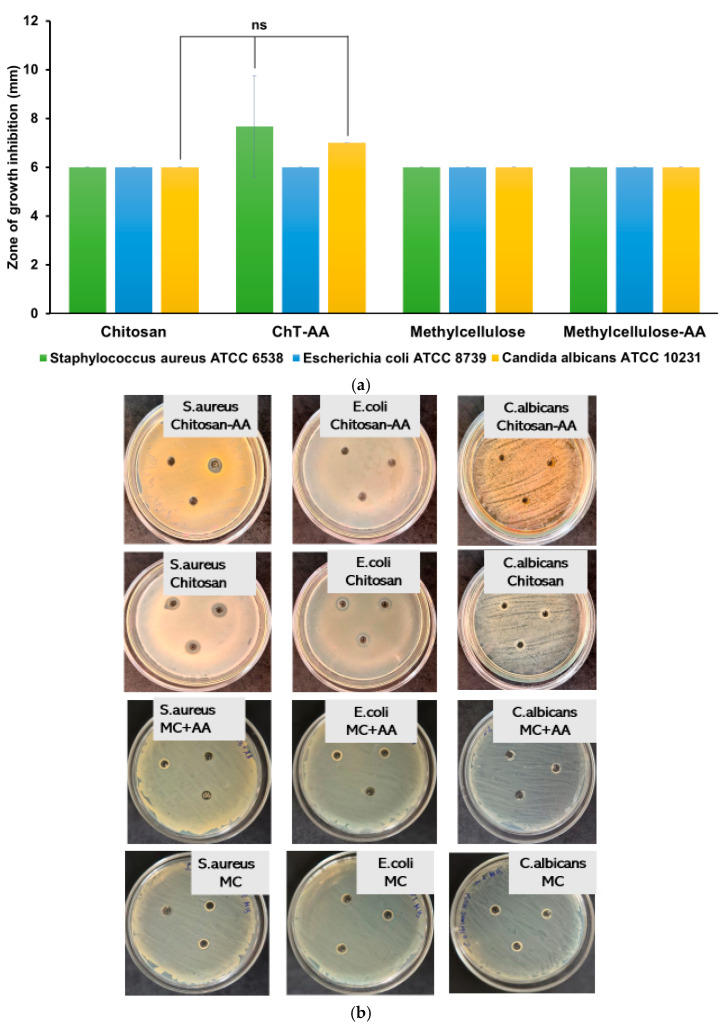
Antimicrobial activity of gel formulations evaluated by the disk diffusion assay (DDA). The inhibition zone diameters (mm) for *E. coli*, *S. aureus*, and *C. albicans* (**a**); and the photographic insets illustrate representative agar plates after 24 h of incubation at 37 °C for the corresponding microorganisms (**b**). Statistically significant differences are given as: ns—no significance.

The antimicrobial activity of gel compositions containing chitosan, conjugate and 4-anisaldehyde was evaluated against *Escherichia coli* (Gram-negative), *Staphylococcus aureus* (Gram-positive), and *Candida albicans* using the disk diffusion assay (DDA) (Figure 7). According to ^1^H NMR spectroscopy, the degree of conjugation between chitosan and 4-anisaldehyde in the ChT–AA (1:3) derivative was quantified, and based on this value, a methylcellulose (MC) gel containing free 4-anisaldehyde (MC–AA) was prepared, where the aldehyde concentration corresponded to its molar fraction in the conjugate.

As shown in Figure 7, both the chitosan/MC and ChT–AA(1:3)/MC gels exhibited limited inhibition zones with no statistically significant differences (*p* > 0.05) compared to unmodified chitosan. These results suggest that under the tested conditions, the release and diffusion of 4-anisaldehyde—either free or conjugated—were insufficient to produce a pronounced antimicrobial response, though a minor trend toward improved inhibition with the presence of 4-anisaldehyde was observed. This may be attributed to the restricted diffusivity of the active component from the polymeric matrix within the agar, limiting its availability at the microbial interface.

**Figure 8:**
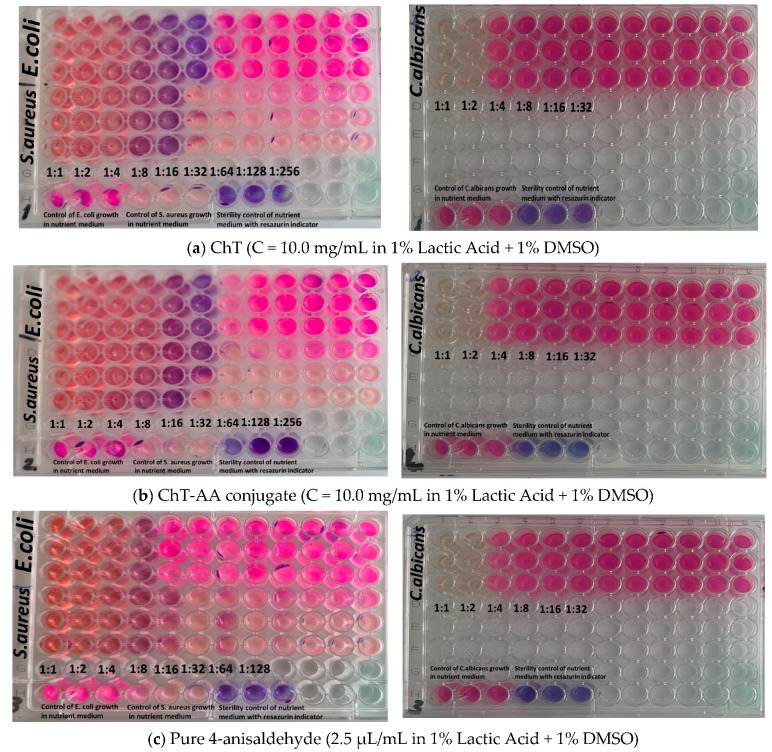

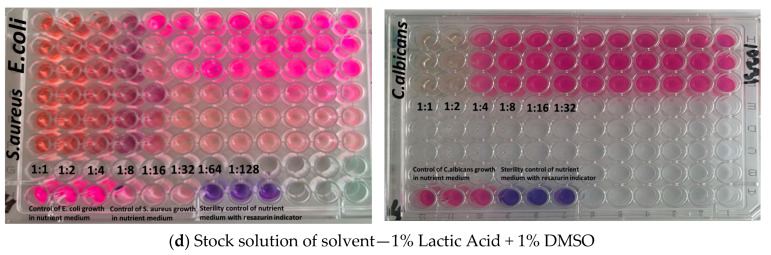
Images illustrating the evaluation of antimicrobial activity of the tested formulations using the serial dilution assay: (**a**) ChT (10.0 mg/mL, 1% lactic acid + 1% DMSO); (**b**) ChT-AA conjugate (10.0 mg/mL, 1% lactic acid + 1% DMSO); (**c**) pure 4-anisaldehyde (2.5 μL/mL, 1% lactic acid + 1% DMSO); and (**d**) solvent control (1% lactic acid + 1% DMSO).

Subsequent experiments were conducted in solution using the resazurin-based broth microdilution assay and results are presented in Figure 8. Table 4 summarises the minimum inhibitory concentrations (MICs) of the tested samples against *Escherichia coli* ATCC 8739, *Staphylococcus aureus* ATCC 6538, and *Candida albicans* ATCC 10231, determined using these data. Four formulations were evaluated: chitosan (ChT), chitosan conjugated with 4-anisaldehyde (ChT-AA), pure 4-anisaldehyde, and the solvent control (1% lactic acid + 1% DMSO).

Both ChT and ChT–AA exhibited identical MIC values (0.313 mg/mL) against *E. coli* and 1.250 mg/mL against *C. albicans*, indicating that chemical modification did not enhance antimicrobial potency under the tested conditions. The MIC values determined for chitosan are consistent with previously reported ranges for chitosan-based materials [35,36]. However, the MIC of ChT–AA against *S. aureus* (0.313 mg/mL) was two-fold lower than that of unmodified ChT (0.625 mg/mL), suggesting improved activity against Gram-positive bacteria. Free 4-anisaldehyde demonstrated higher activity against *S. aureus* (MIC = 0.175 mg/mL) but weaker inhibition of *E. coli* and *C. albicans* (MICs = 0.351 mg/mL), reflecting its limited antimicrobial spectrum. The stock solution of 1% lactic acid + 1% DMSO showed no inhibitory activity even at the lowest dilution (1:8), confirming that the observed effects were due to the active samples rather than the solvent system.

## 4. Conclusions

The modification of chitosan with 4-anisaldehyde (ChT-AA) via Schiff base formation was successfully achieved, which was confirmed using FTIR and ^1^H NMR spectroscopy. TGA and DSC analysis of these conjugates indicated changes in their thermal properties compared to the parent chitosan.

Gel formulations were prepared based on ChT-AA and chitosan with methylcellulose as a gelling agent. These gels exhibited thixotropic behaviour suitable for topical biomedical formulations.

The disk diffusion assay revealed no detectable antimicrobial activity of either ChT–AA(1:3)/MC or ChT/MC gels, suggesting limited diffusion of both 4-anisaldehyde released from the conjugate and chitosan itself through the agar medium. In contrast, the broth microdilution test demonstrated measurable inhibitory activity, particularly for ChT and ChT-AA conjugate formulations. Overall, conjugation with 4-anisaldehyde provided a modest improvement in activity against S. aureus, while no enhancement was observed against *E. coli* and *C. albicans*. These findings highlight the importance of diffusion properties in the antimicrobial performance of chitosan-based gels and suggest potential for further optimisation through controlled release design.

## Figures and Tables

**Table 1:** Reactant ratios used for the synthesis of ChT-AA conjugates.

Molar Ratio of [Chitosan]:[AA]	Weight of Chitosan, g	Weight of AA, g
1.6:1	1.00	0.25
1:3	1.00	1.26
1:1	1.00	0.42

**Table 2:** Results of thermal analysis of Chitosan and ChT-AA.

Sample	Water Content in the Samples, %	T (Onset of Degradation), °C ^1^	Tmax, °C ^1^	T_g_, °C ^2^	Temperature of Exothermic Event, °C ^2^	Temperature of Endothermic Event, °C ^2^	Residual Weight at 600 °C, %
Chitosan	10	200	350	117	314	76	13
ChT-AA	0.5	230	400	122.5	309	74	29.7

^1^ determined by the TGA method. ^2^ determined by the DSC method.

**Table 3:** Results of rheological property analysis of the Chitosan and ChT-AA gel samples.

Spindle Rotation Speed, rpm	Shear Rate, γ, s^−1^	Gel with ChT-AA				Gel with Chitosan			
	(*γ* = *K·RPM*)	Ascending		Descending		Ascending		Descending	
		Shear Stress, τ, mPa	Viscosity, η, mPa·s	Shear Stress, τ, mPa	Viscosity, η, mPa·s	Shear Stress, τ, mPa	Viscosity, η, mPa·s	Shear Stress, τ, mPa	Viscosity, η, mPa·s
		(*τ* = *η·γ*)		(*τ* = *η·γ*)		(*τ* = *η·γ*)		(*τ* = *η·γ*)	
0.5	0.5	3815	7630 ± 1145	3715	7430 ± 1115	30,916.5	61,833 ± 9275	26,137.5	52,275 ± 7841
50	50	176,900	3538 ± 531	155,600	3112 ± 467	516,100	10,322 ± 1548	138,200	2764 ± 415
100	100	284,000	2840 ± 426	252,300	2523 ± 378	586,300	5863 ± 879	205,000	2050 ± 308
150	150	376,050	2507 ± 376	330,750	2205 ± 331	655,200	4368 ± 655	277,500	1850 ± 278
200	200	453,000	2265 ± 340	400,600	2003 ± 300	684,600	3423 ± 513	331,000	1655 ± 248
250	250	535,500	2142 ± 321	460,750	1843 ± 276	719,500	2878 ± 432	370,000	1480 ± 222
300	300	599,100	1997 ± 300	519,000	1730 ± 260	764,100	2547 ± 382	416,700	1389 ± 208
350	350	638,750	1825 ± 274	571,550	1633 ± 245	793,800	2268 ± 340	453,600	1296 ± 194
400	400	682,800	1707 ± 256	620,400	1551 ± 233	816,000	2040 ± 306	517,600	1294 ± 194
450	450	733,050	1629 ± 244	668,700	1486 ± 223	880,650	1957 ± 294	556,200	1236 ± 185
500	500	773,500	1547 ± 232	712,500	1425 ± 214	874,000	1748 ± 262	584,500	1169 ± 175
550	550	806,300	1466 ± 220	753,500	1370 ± 206	902,000	1640 ± 246	620,950	1129 ± 169
600	600	843,600	1406 ± 211	788,400	1314 ± 197	891,600	1486 ± 223	672,000	1120 ± 168
650	650	880,100	1354 ± 203	825,500	1270 ± 191	925,600	1424 ± 214	708,500	1090 ± 164
700	700	910,700	1301 ± 195	861,000	1230 ± 185	935,200	1336 ± 200	728,700	1041 ± 156
750	750	918,000	1224 ± 184	897,750	1197 ± 180	947,250	1263 ± 189	771,000	1028 ± 154
800	800	948,000	1185 ± 178	948,000	1185 ± 178	999,200	1249 ± 187	999,200	1249 ± 187

**Table 4:** Minimum inhibitory concentrations (MICs) of tested samples against reference microbial strains.

Sample	Minimum Inhibitory Concentration (MIC), mg/mL		
	*E. coli* ATCC 8739	*S. aureus* ATCC 6538	*C. albicans* ATCC 10231
1. ChT (C = 10 mg/mL in 1% Lactic Acid + 1% DMSO)	0.313	0.625	1.250
2. ChT-AA (C = 10 mg/mL in 1% Lactic Acid + 1% DMSO)	0.313	0.313	1.250
3. Pure 4-anisaldehyde (2.5 μL/mL in 1% Lactic Acid + 1% DMSO)	0.351	0.175	0.351
4. Stock solution of solvent—1% Lactic Acid + 1% DMSO (11 mg/mL of DMSO in 1 % Lactic acid + H_2_O)	1.375	0.6875−1.375	1.375

## Data Availability

The original contributions presented in this study are included in the article. Further inquiries can be directed to the corresponding author.

## Abbreviations

The following abbreviations are used in this manuscript:

ChT	chitosan
AA	anisaldehyde
ChT-AA	chitosan-anisaldehyde conjugate
FTIR	Fourier-transform infrared spectroscopy
^1^H NMR	proton nuclear magnetic resonance
DSC	Differential scanning calorimetry
TGA	Thermogravimetric analysis
NaFl	sodium fluorescein
T_g_	glass transition temperature
